# The bradykinin system in stress and anxiety in humans and mice

**DOI:** 10.1038/s41598-019-55947-5

**Published:** 2019-12-19

**Authors:** Ari Rouhiainen, Natalia Kulesskaya, Marie Mennesson, Zuzanna Misiewicz, Tessa Sipilä, Ewa Sokolowska, Kalevi Trontti, Lea Urpa, William McEntegart, Suvi Saarnio, Petri Hyytiä, Iiris Hovatta

**Affiliations:** 1 Molecular and Integrative Biosciences Research Program, University of Helsinki, Helsinki, Finland; 2 Department of Psychology and Logopedics, Medicum, University of Helsinki, Helsinki, Finland; 3 SleepWell Research Program, Faculty of Medicine, University of Helsinki, Helsinki, Finland; 4 Neuroscience Center, Helsinki Institute of Life Science HiLIFE, University of Helsinki, Helsinki, Finland; 5 Department of Pharmacology, Medicum, University of Helsinki, Helsinki, Finland

**Keywords:** Behavioural genetics, Gene expression, Anxiety, Physiology

## Abstract

Pharmacological research in mice and human genetic analyses suggest that the kallikrein-kinin system (KKS) may regulate anxiety. We examined the role of the KKS in anxiety and stress in both species. In human genetic association analysis, variants in genes for the bradykinin precursor (*KNG1*) and the bradykinin receptors (*BDKRB1* and *BDKRB2*) were associated with anxiety disorders (p < 0.05). In mice, however, neither acute nor chronic stress affected B1 receptor gene or protein expression, and B1 receptor antagonists had no effect on anxiety tests measuring approach-avoidance conflict. We thus focused on the B2 receptor and found that mice injected with the B2 antagonist WIN 64338 had lowered levels of a physiological anxiety measure, the stress-induced hyperthermia (SIH), vs controls. In the brown adipose tissue, a major thermoregulator, WIN 64338 increased expression of the mitochondrial regulator *Pgc1a* and the bradykinin precursor gene *Kng2* was upregulated after cold stress. Our data suggests that the bradykinin system modulates a variety of stress responses through B2 receptor-mediated effects, but systemic antagonists of the B2 receptor were not anxiolytic in mice. Genetic variants in the bradykinin receptor genes may predispose to anxiety disorders in humans by affecting their function.

## Introduction

The kallikrein-kinin system (KKS) was first discovered seven decades ago in studies on human urine due to its hypotensive effect^1^. The KKS has many central and peripheral physiological functions mediated by peptides cleaved from kininogen. Kininogen-derived peptides bradykinin and kallidin bind to bradykinin receptor B2 (BDKRB2) whereas their degradation products des-Arg-bradykinin and des-Arg-kallidin bind to bradykinin receptor B1 (BDKRB1). BDKRB2 is constitutively expressed in many cell types, while BDKRB1 is normally expressed at a relatively low levels in most tissues, but can be strongly up-regulated, for example due to inflammation^2^. Bradykinin activates several second messenger systems and thereby regulates blood-brain barrier permeability, blood pressure, pain perception, release of glutamate from astrocytes, neuronal differentiation and nitric oxide production^3^. The KKS has been implicated in the aetiology of many brain diseases, including epilepsy, Alzheimer’s disease, traumatic brain injury and multiple sclerosis^4^.

Genetic associations have the potential to lead to understanding of the biochemical, cellular and physiological mechanisms that underlie common diseases, including neuropsychiatric disorders such as the anxiety disorders. These include panic disorder, social phobia, specific phobias, and generalized anxiety disorder, and they were the most common mental illnesses in the EU in 2010, with a prevalence of 14%^5^. Human genetic data support the involvement of the KKS in the aetiology of anxiety disorders. A functional genetic variant within the promoter region of *BDKRB2* has been associated with panic disorder in a candidate gene study of 306 modulators of neurotransmitter systems^6^. Moreover, another promoter variant of *BDKRB2* was the most significantly associated SNP in a Japanese genome-wide association study meta-analysis of panic disorder^7^. Angiotensin converting enzyme (ACE) is a major bradykinin inactivating enzyme. Its inhibitors are common antihypertensive agents. In patients with posttraumatic stress disorder (PTSD), variants in *ACE* associate both with the severity of PTSD symptoms, and the effectiveness of ACE inhibitors to attenuate them^8^. It is not known, however, whether this effect is mediated by the KKS.

Rodent studies also support the involvement of the KKS in anxiety. Intracerebroventricular (i.c.v.) injection of bradykinin into the rat brain increases anxiety-like behaviour and reduces social interaction^9^. Periaqueductal grey (PAG) is a midbrain structure involved in controlling defence responses and panic-like behaviour^10^. Bradykinin injection to PAG is panicolytic, an effect mediated by the BDKRB2 and *μ*–opioid receptors^11,12^.

In addition to genetic factors, the predisposition to anxiety disorders is also affected by environmental risk factors, such as stress^13^. The adrenal gland releases catecholamines and glucocorticoids in response to stress. Bradykinin participates in these processes by functioning as a secretagogue for catecholamines in the adrenal medulla^14^, and by affecting glucocorticoid secretion. Chronic downregulation of endogenous bradykinin levels with kallikrein inhibitors enhances corticosterone secretion during mild stress^15^, and chronic administration of exogenous bradykinin in turn downregulates plasma corticosterone levels^16^. How these bradykinin-related changes in corticosterone levels are related to stress-associated behaviours, such as anxiety, is not known.

The role of the KKS in neurological diseases is well-established^17^, but its role in psychiatric illnesses is less well-known. We hypothesized that the KKS system may regulate stress- and anxiety-related behaviours. We first set to replicate the previously observed human genetic association findings of *BDKRB2* and to extend our study to the other KKS genes, the *KNG1* (Kininogen 1, coding for the bradykinin peptide) and the *BDKRB1*. Since both strong acute stressful events and chronic stress, especially in the form of psychosocial stress, are well-known risk-factors for anxiety disorders, we then investigated whether acute or chronic stress affects brain bradykinin receptor expression. We studied both gene and protein levels in the mouse brain regions known to be involved in anxiety and stress responses because of potential differences in the temporal pattern and post-transcriptional regulation of these molecules. Furthermore, we tested the effect of BDKRB1 and BDKRB2 antagonists on mouse anxiety-like behaviour and physiological stress responses.

## Results

### Association analysis of KKS genes and anxiety disorders

To investigate whether genetic variants in the KKS genes associate with anxiety disorders, we genotyped SNPs from *KNG1*, *BDKRB1*, and *BDKRB2* in anxiety disorder cases (n = 321) and carefully matched controls (n = 653) from the Finnish population-based Health 2000 Survey. Of the 21 analysed *KNG1* SNPs, 17 *BDKRB1* SNPs, and 20 *BDKRB2* SNPs, 5, 8, and 1 associated with anxiety disorders at the nominal p < 0.05 level, respectively (Table 1).

**Table 1:** SNPs showing evidence for association at p < 0.05 level in allele or genotype-based test.

Gene	SNP	Alleles [A1/A2]	Group	Allele frequencies		Allelic LRT p-value	Genotype frequences			Genotypic LRT p-value
				A1	A2		A1/A1	A1/A2	A2/A2	
*BDKRB1*	rs2069613	C/T	Cases	0.107	0.893	0.0220	0.009	0.195	0.796	0.0753
			Controls	0.143	0.857		0.020	0.246	0.734	
	rs4905475	C/G	Cases	0.136	0.864	**0.0006**	0.016	0.241	0.743	**0.0007**
			Controls	0.083	0.917		0.014	0.138	0.848	
	rs11622768	C/T	Cases	0.864	0.136	**0.0003**	0.744	0.240	0.016	**0.0006**
			Controls	0.917	0.083		0.848	0.138	0.014	
	rs10147171	C/T	Cases	0.137	0.863	**0.0002**	0.016	0.242	0.742	**0.0001**
			Controls	0.084	0.916		0.014	0.140	0.846	
	rs10137303	A/G	Cases	0.863	0.137	**0.0008**	0.742	0.242	0.016	**0.0009**
			Controls	0.918	0.082		0.850	0.136	0.014	
	rs11628515	A/T	Cases	0.136	0.864	**0.0005**	0.016	0.241	0.743	**0.0006**
			Controls	0.083	0.917		0.014	0.138	0.848	
	rs4349080	A/G	Cases	0.197	0.803	0.0139	0.054	0.287	0.659	0.0332
			Controls	0.249	0.751		0.069	0.361	0.571	
	rs945034	C/T	Cases	0.540	0.460	0.0353	0.299	0.481	0.220	0.0900
			Controls	0.590	0.410		0.345	0.491	0.165	
*BDKRB2*	rs4900311	A/G	Cases	0.454	0.546	0.0096	0.208	0.492	0.300	0.0323
			Controls	0.517	0.483		0.263	0.509	0.229	
	rs1799722	C/T	Cases	0.595	0.405	0.7747	0.328	0.533	0.139	0.0296
			Controls	0.588	0.412		0.364	0.448	0.188	
	rs2069575	A/G	Cases	0.117	0.883	0.0105	0.019	0.196	0.785	0.0239
			Controls	0.160	0.840		0.023	0.274	0.703	
	rs5225	C/T	Cases	0.139	0.861	**0.0022**	0.016	0.246	0.738	0.0041
			Controls	0.092	0.908		0.014	0.156	0.830	
	rs2069596	A/G	Cases	0.151	0.849	0.0080	0.019	0.263	0.717	0.0112
			Controls	0.108	0.892		0.019	0.179	0.803	
*KNG1*	rs13315296	C/T	Cases	0.519	0.481	0.0404	0.286	0.465	0.248	0.0917
			Controls	0.468	0.532		0.223	0.491	0.287	

P-values surviving gene-wide Bonferroni correction for multiple testing are shown in bold.

### The effect of acute stress on KKS-related gene and protein expression in the mouse brain

**Figure 1:**
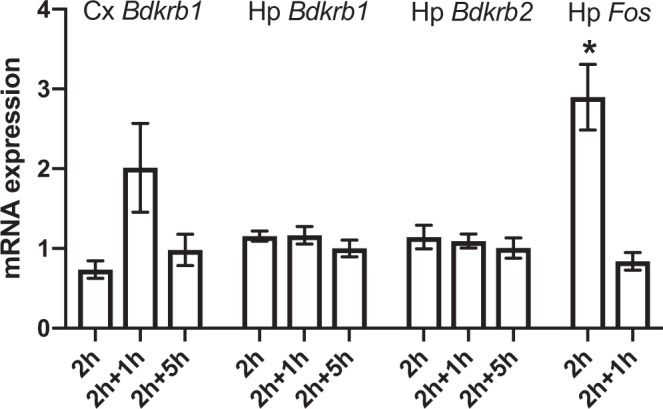
The effect of acute stress on mouse brain bradykinin receptor gene expression. mRNA levels of *Bdkrb1*, *Bdkrb2* and *Fos* in cortex (Cx) and hippocampus (Hp) directly (2 h), 1 h (2 h + 1 h) or 5 h (2 h + 5 h) after 2 h restraint stress in C57BL/6NCrl mice. N = 8 in Cx *Bdkrb1*, N = 7–12 in Hp *Bdkrb1* and Hp *Bdkrb2*, and N = 3 in Hp *Fos*. The mRNA expression level of the control group was defined as 1. Bars represent the mean ± SEM of the normalised mRNA expression level. *p < 0.05 compared to non-stressed control mice.

To investigate the effect of acute stress on the gene expression levels of the bradykinin receptors, we subjected C57BL/6NCrl (B6) mice to restraint stress for 2 hours, and allowed them to recover for 0, 1, or 5 hours, after which we collected tissue from the entire hippocampus and cortex. Control mice did not experience restraint stress. Acute stress did not significantly affect hippocampal gene expression levels of *Bdkrb1* or *Bdkrb2*. In the cortex, there was a trend for higher gene expression levels of *Bdkrb1* at the 2 h + 1 h time point (two tailed t-test p = 0.107) compared to controls (Fig. 1). *Bdkrb2* expression levels were below detection level in the cortex. The expression level of the positive control gene, FBJ osteosarcoma oncogene *Fos*^18^ was significantly upregulated by stress (p = 0.011) (Fig. 1).

**Figure 2:**
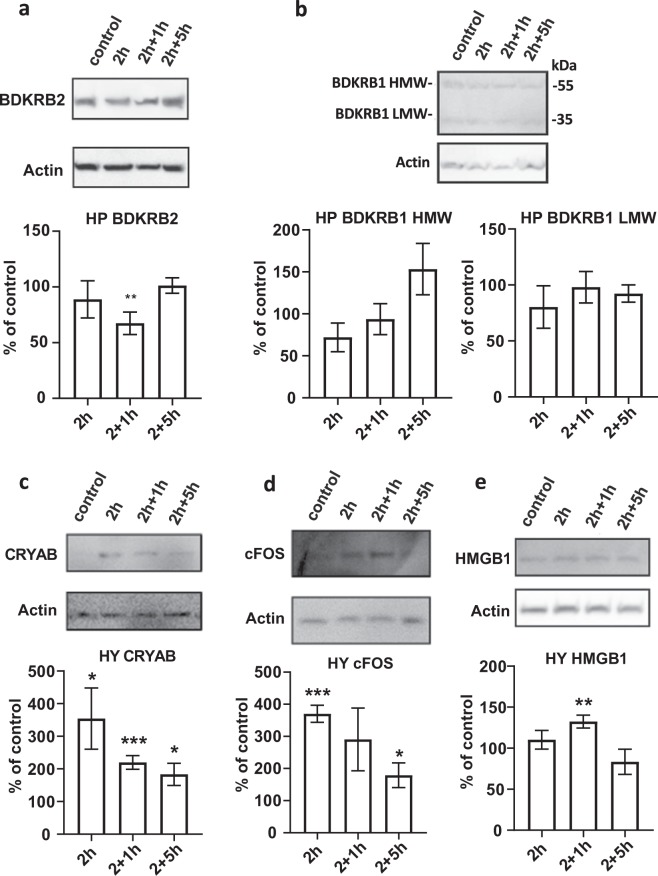
The effect of acute stress on bradykinin receptor protein expression in the hippocampus. We measured normalized protein expression levels of BDKRB1 and BDKRB2 using Western blot in hippocampus (**a**,**b**) and control stress-associated genes in hypothalamus (**c**–**e**) directly (2 h), 1 h (2 h + 1 h) or 5 h (2 h + 5 h) after 2 h restraint stress in C57BL/6NCrl mice. BRKRB1 HMW = bradykinin receptor B1 high molecular weight, BRKRB1 LMW = bradykinin receptor B1 low molecular weight, CRYAB = α-B-crystallin, FOS = FBJ osteosarcoma oncogene, HMGB1 = high mobility group B1. N = 3–5 in each experiment. Bars represent mean ± SEM. *p < 0.05, **p < 0.01, ***p < 0.001compared to non-stressed control mice. For uncropped blots, see Supplemental Fig. 1.

We next used the Western blot method to assess whether acute stress affects the protein levels of bradykinin receptors in the mouse hippocampus. We subjected B6 mice to restraint stress as described above and collected hippocampus and hypothalamus (control) tissue for protein analysis. Protein levels of BDKRB2 were significantly downregulated in mice that underwent 2 h restraint stress and 1 h recovery (p = 0.005, Fig. 2a). The protein levels of BDKRB1 were not affected by stress (Fig. 2b). As positive controls for the stress experiment, we used well-established markers α-B-crystallin^19,20^, FOS^18,21^, and HMGB1^22,23^, all of which were upregulated by stress in the hypothalamus, as expected (Fig. 2c–e).

### Effect of chronic stress on KKS gene expression

**Figure 3:**
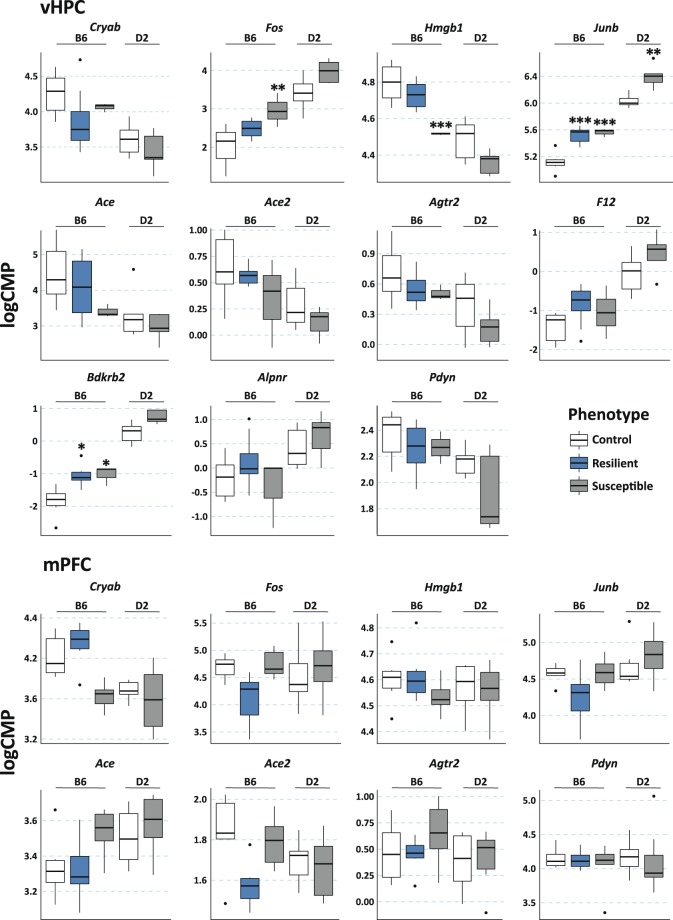
Expression levels of KLK-related genes after chronic social defeat stress in mice. *Agtr2*, *Bdkrb1*, *Klk1*, *Klk12*, *Klkb1, Kng1* and *Kng2* were included in the analysis but their expression levels were below the detection level in both brain regions. *F12*, *Bdkrb2* and *Aplnr* expression levels were below the detection level in the mPFC. LogCPM = normalised expression level in log Counts Per Million from RNA-sequencing. P-values (from moderated t-test for differential expression) are corrected for multiple testing with Benjamini-Hochberg method. Box plots show minimum, 1st quartile, median, 3rd quartile, and maximum expression value for each group. Data outliers (values > 1.5 interquartiles from the IQR range) are depicted by a dot. vHPC = ventral hippocampus, mPFC = medial prefrontal cortex, B6 = C57BL/6NCrl, D2 = DBA/2NCrl. N of mice: mPFC (B6: controls 6, resilient 6, susceptible 6; D2: controls 6, susceptible 8) and vHPC (B6: controls 6, resilient 8, susceptible 3; D2: controls 6, susceptible 5). *p < 0.05, **p < 0.01, ***p < 0.001 compared to the same-strain control group.

Chronic social defeat stress (CSDS) is a well-established psychosocial stress model in mice. It leads to increased social avoidance behaviour in susceptible mice, while some animals are resilient to the behavioural effects of stress^24^. We analysed gene expression levels in the ventral hippocampus and medial prefrontal cortex after 10 days of CSDS using RNA-sequencing^25^. We extracted data for KKS genes (*Bdkrb1*, *Bdkrb2, F12, Klk1*, *Klk12*, *Klkb1, Kng1 and Kng2*), genes related to the KKS genes (*Ace*, *Ace2*, *Agtr2*, *Aplnr* and *Pdyn*), and known marker genes of stress (*Cry*, *Fos*, *Junb* and *Hmgb1*). Figure 3 shows the expression levels of those genes that were reliably detected in the two studied mouse strains, innately anxious DBA/2NCrl (D2) and non-anxious B6. In the ventral hippocampus, *Bdkrb2* and *Jun* expression levels were significantly higher in both stress-susceptible (p = 0.011 and 0.00037, respectively) and resilient (p = 0.012 and 0.00016, respectively) B6 mice compared to controls. In addition, *Jun* expression levels were higher in the susceptible D2 mice compared to controls (p = 0.0023). *Fos* expression levels were higher (p = 0.0025) and *Hmgb1* levels lower (p = 0.00090) in the susceptible B6 mice. None of the studied genes was differentially expressed in the medial prefrontal cortex after CSDS (Fig. 3).

### Behavioural effects of pharmacological targeting of bradykinin receptors in mice

**Figure 4:**
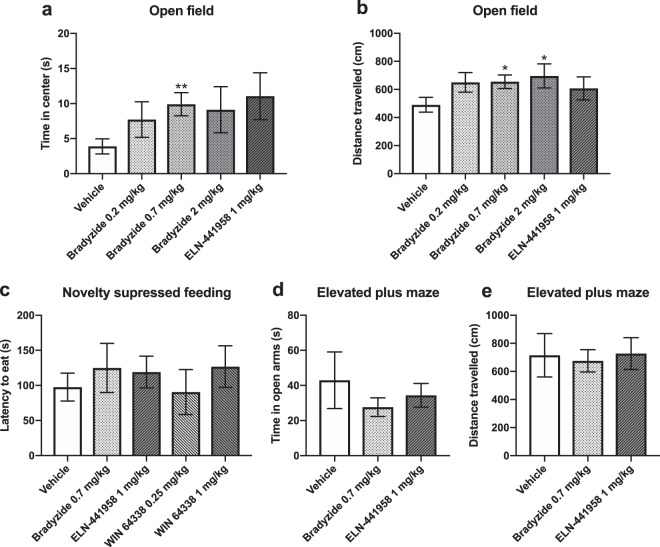
The effect of bradykinin receptor antagonists on anxiety-like behaviour. We injected DBA/2NCrl mice subcutaneously with B1 receptor non-peptide receptor antagonist ELN-441958 or B2 receptor non-peptide antagonists bradyzide or WIN 64338 and carried out behavioural testing 30 min later in the open field test (N = 8–12 per group) (**a**,**b**), in the novelty suppressed feeding test (N = 8–10 per group) (**c**) and in the elevated plus maze test (N = 7–9 per group) (**d**,**e**). Bars represent the mean ± SEM. *p < 0.05, **p < 0.001 compared to the vehicle (PBS/0.05% DMSO) group.

To investigate whether blocking bradykinin receptor function affects anxiety-like behaviour, we injected D2 mice with bradykinin receptor B1 or B2 antagonists, followed by behavioural testing. We selected physiological doses using prior mouse literature^26–29^. We first studied the effect of subcutaneously (s.c.) injected B1 receptor non-peptide antagonist ELN-441958 and B2 receptor non-peptide antagonists bradyzide or WIN 64338 on mouse behaviour in the open field, novelty suppressed feeding and elevated plus maze tests. In the open field test, mice that received 0.7 mg/kg dose of bradyzide spent more time in the centre zone than controls, suggesting reduced anxiety-like behaviour (p = 0.0058, Fig. 4a). However, mice that received higher doses of bradyzide also had higher locomotor activity than controls, as indicated by longer distance travelled in the open field (bradyzide 0.7 mg/kg p = 0.031, bradyzide 2.0 mg/kg p = 0.044; Fig. 4b). We did not observe differences between antagonist and control injected mice in the novelty suppressed feeding or elevated plus maze tests (Fig. 4c–e).

### The effect of pharmacological targeting of the bradykinin B2 receptor on mouse stress-induced behaviour

**Figure 5:**
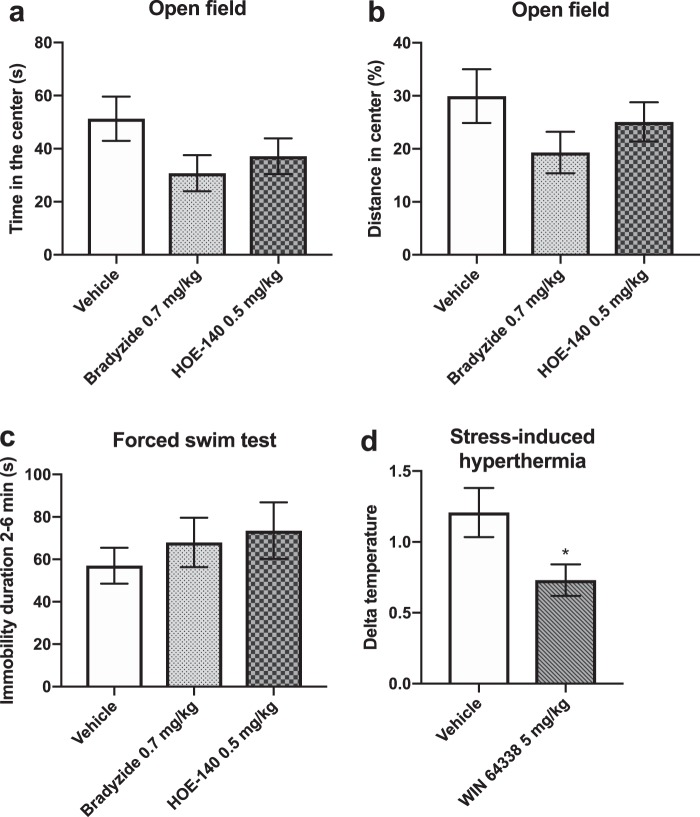
The effect of bradykinin B2 receptor antagonists on mouse stress-related behaviour. DBA/2NCrl mice were injected intraperitoneally with drug or vehicle, subjected to 30 min restraint stress and let recover for 1 h, followed by behavioural testing in the open field (**a**,**b**) or forced swim test. (**c**) N = 8–9 per group. Effect of WIN 64338 on body temperature in stress induced hypothermia (SIH) model. (**d**) Temperature difference ∆T (T_2_-T_1_) is shown. N = 13 per group. Bars represent the mean ± SEM. *p < 0.05 compared to the vehicle control group.

Since acute or chronic stress did not affect gene or protein expression of BDKRB1, nor did the BDKRB1 receptor antagonist influence anxiety-like behaviour, we concentrated on the BDKRB2. We next investigated the B2 receptor antagonists in more detail concentrating on their effects on stress-induced anxiety-like and despair behaviours. For this purpose, we injected D2 mice i.p. with the B2 receptor peptide antagonist HOE-140, the small molecule antagonist bradyzide or vehicle. We then immediately subjected the mice to 30 min restraint stress and let them recover for 1 h before testing for anxiety-like behaviour in the open field test and despair behaviour in the forced swim test. We did not detect any differences in the behaviour of the drug-injected mice compared to the controls (Fig. 5a–c), although there was a trend for shorter open field centre time in mice that received 0.7 mg/kg dose of bradyzide compared to vehicle controls (p = 0.075, Fig. 5a).

The stress-induced hyperthermia (SIH) paradigm measures increases in body temperature due to stress caused by handling, possible injection and rectal body temperature measurements of mice, and it is therefore considered a physiological test of anxiety-like behaviour^30^. We measured rectal body temperature (T_0_) and injected D2 mice with the small molecule B2 antagonist WIN 64338 (5 mg/kg, i.p.) or vehicle. Body temperature was measured again 60 min (T_1_) and 70 min (T_2_) after T_0_. The increase in body temperature between 60 and 70 min (T_2_-T_1_) was considered the measure of SIH (∆T). WIN 64338 significantly reduced SIH (p = 0.029) (Fig. 5d), indicating an anxiolytic effect in this test that is not confounded by locomotor activity of mice.

### The effect of stress and a B2 receptor antagonist on brown adipose tissue KKS gene expression

Thermogenesis is partly regulated by brown adipose tissue (BAT), and therefore we conducted gene expression analysis in BAT to study i) whether cold stress-induced thermogenesis influences the expression levels of the KKS genes in this tissue, and ii) whether the B2 receptor antagonist WIN 64338 affects BAT gene expression during SIH.

**Figure 6:**
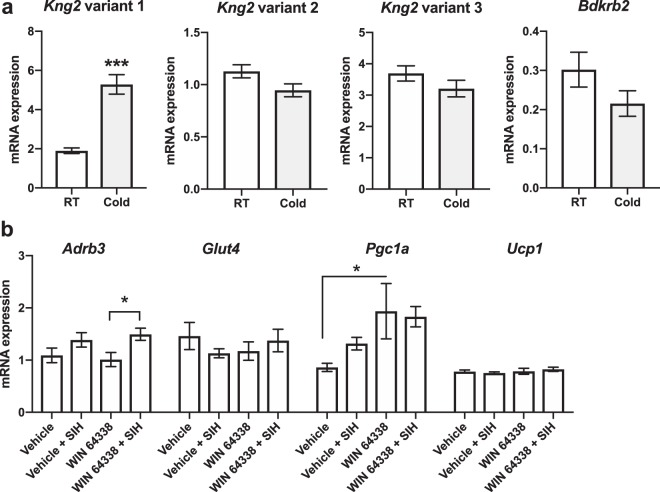
Stress-induced changes in the brown adipose tissue (BAT) gene expression levels. (**a**) Expression levels of *Kng2* variants and *Bdkrb2* transcripts in the interscapular BAT of cold-stressed and control C57BL/6NCrl mice (N = 11–13 per group). The y-axis shows the relative mRNA expression levels. Due to different amounts of input cDNA, expression levels of the different transcripts are not readily comparable. ***p < 0.001 compared to the room temperature (RT) control. (**b**) The effect of stress-induced hyperthermia (SIH) and WIN 64338 on BAT gene expression levels of DBA/2NCrl mice. N = 6–9 per group. For statistical significances, see the main text. Bars represent the mean ± SEM. *p < 0.05 calculated by Bonferroni post-hoc comparison.

In the cold stress experiment, we subjected B6 mice to 4 °C temperature for 4 hours, after which we collected interscapular BAT, and analysed the expression levels of kininogen transcript variants and bradykinin receptors. We analysed expression of both *Kng1* and *Kng2*, as mice have two *Kng* genes expressing identical bradykinin peptides. The *Kng2* variant 1 coding for the high molecular weight kininogen (HMWK) was upregulated by cold stress (p < 0.001) whereas the expression levels of the other *Kng2* variants and the *Bdkrb2* remained unaltered (Fig. 6a). The expression levels of the *Kng1* variants and the *Bdkrb1* were below the detection level.

Next, to investigate whether BAT is activated by SIH and whether BDKRB2 modulates this effect, we analysed the expression levels of BAT activation marker genes by qPCR after i.p. injection of a B2 antagonist (WIN 64338, 5 mg/kg, i.p.) or vehicle and SIH or non-stressed controls (that experienced mild stress in the form of an i.p. injection) in D2 mice. We observed a significant stress effect in *Adrb3* (adrenoceptor 3) expression (2-way ANOVA for stress and drug effects, stress effect p = 0.007), *Adrb3* expression being higher after SIH in WIN 64338-injected mice compared to non-stressed mice that received WIN 64338 (post-hoc WIN 64338 vs WIN 64338 + SIH, p = 0.013) (Fig. 6b). There was a significant drug effect in *Pgc1a* (PPARG coactivator 1 alpha) expression levels (2-way ANOVA, drug effect p = 0.010), with the higher expression levels in WIN 64338 group compared to the vehicle group (post-hoc WIN 64338 vs vehicle p = 0.015). The expression levels of *Glut4* (glucose transporter 4) and *Ucp1* (uncoupling protein 1) did not differ between the groups (Fig. 6b).

## Discussion

We used multiple approaches to investigate whether the KKS regulates stress-related phenotypes and anxiety disorders. We took advantage of a Finnish population-based genetic sample with well-characterized anxiety disorder cases and carefully matched controls. Furthermore, to clarify experimentally the role of the KKS in stress and anxiety, we used both acute and chronic mouse stress models and investigated if the expression of KKS genes and proteins are affected by stress, and whether the bradykinin receptor antagonists influence mouse behaviour.

We established a role for the KKS in the genetic predisposition to anxiety disorders in humans, and in physiological stress response in mice. BDKRB2 protein levels were decreased in the mouse hippocampus by acute stress while *Bdkrb2* gene expression levels were increased after chronic psychosocial stress, a major risk factor for anxiety disorders. Although we did not detect an anxiolytic effect of bradykinin receptor antagonists in anxiety tests measuring approach-avoidance conflict, a BDKRB2 antagonist was anxiolytic in the SIH model. We further demonstrated that a specific *Kng* transcript was upregulated by cold stress in BAT. Of the studied BAT activity markers, *Adrb3* was induced by stress in mice that received a BDKRB2 antagonist, while the antagonist itself induced expression of *Pgc1a*, a key regulator of energy metabolism.

In human genetic studies, the bradykinin system genes have previously been associated with panic disorder and obsessive-compulsive disorder^6^. Therefore, we systematically selected a comprehensive set of SNPs from the *KNG1, BDKRB1*, and *BDKRB2* genes and genotyped them in a well-characterized Finnish anxiety disorder case-control sample, representative of the Finnish population^31^. We found the strongest evidence for association to the *BDKRB1* gene, with five SNPs associating at p < 0.001 level. These SNPs are located in the upstream or intronic regions of the gene. In addition, three SNPs within the *BDKRB2* gene showed association at the p < 0.01 level, one of which, in the 3′UTR region, survived gene-wide multiple testing correction. These SNPs may confer susceptibility to anxiety disorders by affecting the expression levels of the bradykinin receptor genes. Ongoing international genomewide association study efforts in anxiety disorders should clarify how significantly variants in the bradykinin receptor genes influence susceptibility to anxiety disorders, and whether this predisposition is specific to certain anxiety disorder types.

Changes in KKS gene expression have been associated with several neurological and psychiatric diseases. In general, we found that both *Bdkrb1* and *Bdkrb2* were expressed at a very low level in the mouse brain. However, we detected significant downregulation of BDKRB2 protein levels after acute stress, and significant upregulation of mRNA levels after chronic stress in the hippocampus. *Bdkrb2* gene was upregulated in both stress-susceptible and resilient mice, which may indicate a general stress effect rather than an anxiety effect. Many factors could explain the conflicting gene and protein expression differences, and they are most likely related to the timing of the stress experiments. The acute stress involved two-hour restraint stress and one-hour recovery period, while the chronic stress paradigm lasted for 10 days, followed by one-week recovery period. We did not detect differences in the cortex, either after acute or chronic stress. To our knowledge the expression levels of bradykinin or its receptors in patients with anxiety disorders or in animal models of anxiety have not been extensively studied. A Japanese study has linked high plasma bradykinin levels to somatization disorder in two patients whose symptoms were relieved by anti-bradykinin agents^32^. In bipolar disorder, the expression of *KNG1* mRNA is upregulated in the orbitofrontal cortex^33^. Elevated plasma KNG1 protein levels can be detected even before the onset of disease, in neonates who later develop schizophrenia^34^ and in children who later develop psychotic disorder^35^. We have observed in a first episode psychosis study that antipsychotic drug treatment influences *BDKRB1* gene expression levels in blood leukocytes^36^. Thus, changes in the KKS may occur already before disease onset.

Our finding that especially BDKRB2 levels were altered after acute and chronic stress, major risk factors for anxiety disorders, encouraged us to test whether antagonists of BDKRB1 or BDKRB2 affect anxiety-like behaviour in mice. We observed that mice injected s.c. with 0.7 mg/kg of B2 non-peptide antagonist bradyzide spent more time in the centre of the open field arena compared to vehicle injected mice, indicative of reduced anxiety-like behaviour. However, mice injected with the two higher tested concentrations (0.7 or 2.0 mg/kg) showed significantly longer distance travelled during the test compared to the vehicle injected animals, which is suggestive of higher locomotor behaviour. Taking the activity level as a cofactor in the analysis abolished the anxiolytic effect. We also tested anxiety-like behaviour in a test not confounded by activity, the novelty suppressed feeding test, and another commonly used test, the elevated plus maze test. None of the tested antagonists or doses affected anxiety-like behaviour in these tests. These results suggest that bradykinin receptor antagonists do not have clear anxiolytic or anxiogenic effects, but that BDKRB2 antagonists increase locomotor activity.

Because of the significant differences in BDKRB2 expression after acute and chronic stress, we investigated the effect of BDKRB2 non-peptide (bradyzide and WIN 64338) and peptide (HOE-140) antagonists on stress-related behaviours. They did not influence anxiety-like behaviour in the open field test after acute restraint stress or despair-behaviour in the forced swim test. We also tested if blocking BDKRB2 with WIN 64338 affects stress-induced hyperthermia, another model of anxiety-like behaviour not dependent on spontaneous activity. We thought this model was especially relevant due to the known effects of centrally administered bradykinin in fever and hyperthermia^37^. This regulation occurs via both centrally and peripherally expressed bradykinin receptors^38–42^. Hyperthermia induced by central bradykinin is mediated by the hypothalamic paraventricular nucleus^43^. Both prostaglandin-dependent and independent mechanisms are involved in the hyperthermic effects of bradykinin^40,42,44^. We demonstrated that the hyperthermic response of mice that received WIN 64338 prior to the stress was significantly reduced in response to stress compared to vehicle injected mice. This reduced response is likely due to anxiolytic activity as the bradykinin B2 receptor antagonist alone did not have an antipyretic effect. Blood glucose levels are upregulated by stress and bradykinin enhances glucose uptake to skeletal muscles^45,46^. However, glucose uptake by adipocytes of BAT, a key organ in thermogenesis, does not likely mediate the smaller increase in body temperature since the expression levels of *Glut4*, the major glucose transporter of BAT, was not affected by SIH or B2 antagonism.

Mouse kininogen-coding genes *Kng1* and *Kng2* are expressed in adipose tissue and the expression of mouse *Kng1* is regulated in white adipose tissue by cold stress, suggesting a role in thermoregulation^47,48^. We observed that *Kng2* variant 1 is upregulated by cold stress in BAT, which is highly activated by cold exposure. The regulation of adipokines by KKS is suggested by studies linking the polymorphism in *KNG1* to adiponectin levels^49^ and showing the regulation of adipokine levels by bradykinin receptors B1 and B2^50,51^. Furthermore, bradykinin potentiates the uptake of glucose in adipocytes^52^.

We then studied the effect of B2 receptor antagonism on BAT activation. The expression level of *Ucp1*, which codes the protein responsible for the non-shivering thermogenesis of BAT, was not affected by WIN 64338 or SIH. However, WIN 64338 rapidly upregulated *Pgc1a* expression in BAT, suggesting that blocking of B2 receptors may induce BAT mitochondrial biogenesis. Also, in mice that received WIN 64338, expression of adrenoceptor 3, another regulator of BAT thermogenesis, was increased after SIH. Interestingly, post-stress hyperthermia treatment protects against chronic stress-induced anxiety in mouse open field test, suggesting the involvement of the regulation of body temperature in mouse anxiety-like behaviour^53^. Thus, one physiological mechanism of bradykinin in anxiety might be the regulation of body temperature.

It is likely that the different types of stress paradigms we used i.e. acute restraint stress, acute cold stress, and chronic psychosocial stress, impact at least partly different neurobiological substrates^54,55^, and KKS may contribute differentially to the function of these circuits. Moreover, we cannot distinguish between central and peripheral effects of WIN 64338. Activation of peripheral adipocyte glucocorticoid receptor signalling promotes peripheral energy storage while inhibiting the hypothalamic-pituitary-adrenal axis, implicating peripheral adipocyte glucocorticoid receptors as mediators of fat-to-brain signalling^56^. Future research should address whether such mechanisms contribute to the effect of the KKS in SIH. Due to practical reasons, most experiments were carried out in either B6 or D2 mice that have different innate and stress-induced anxiety-like behaviours^25,57^. In the future, studies on the effect of genetic background on KKS function are warranted.

In conclusion, our results establish a role for the KKS in the regulation of stress response and stress-related anxiety-like behaviour. These effects may be modulated physiologically through changes in body temperature, without affecting classical anxiety measures, such as approach-avoidance conflict. Genetic variants in bradykinin receptor genes may further modulate these effects, and thereby contribute to the susceptibility of anxiety disorders and stress-induced anxiety.

## Materials and Methods

### Human sample

The individuals in this study are derived from the Finnish population-based Health 2000 Study carried out in 2000–2001. The ethical review board of the National Institute for Health and Welfare (THL), formerly the National Public Health Institute of Finland (KTL), approved all experimental protocols in this study and the methods used were carried out in accordance with the approved guidelines. Written informed consent was obtained from all participants. We have previously described the anxiety disorder sub-sample in detail^31,58^. Briefly, it consisted of 321 adults diagnosed with an anxiety disorder (n = 282) or an anxiety disorder subthreshold diagnosis (n = 39) according to DSM-IV criteria. Controls (n = 653) were matched for sex, age (+/− 1 year), and hospital catchment area. They lacked anxiety or major mental disorders, had no missing data in anxiety screen questions, and had explicit negative diagnoses for all symptoms of anxiety.

### Human genetic association analysis

We genotyped the SNP markers (see Supplemental Table 1 for genotyped SNPs and information regarding their quality control) using Sequenom MassARRAY technology with iPLEX chemistry (Sequenom, San Diego, CA, USA) according to the manufacturer’s instructions. Primer information is available on request. We performed rigorous quality control as described earlier^59^. We tested for pointwise genetic association by using a conventional 2 × 2 contingency table likelihood-ratio test^60^ (LRT) of independence of SNP allele or genotype counts in cases and controls, as detailed earlier^31^.

### Pharmacological agents

We used the following pharmacological agents: small molecule bradykinin B1 receptor antagonist ELN-441958 (Chembo Pharma, Nanjing, China), bradykinin B2 receptor peptide antagonist HOE-140 (Tocris Bioscience, Bristol, UK), small molecule bradykinin B2 receptor antagonist bradyzide (Sigma-Aldrich, St. Louis, MO, USA), and small molecule bradykinin B2 receptor antagonist WIN 64338 (Santa Cruz Biotechnology Inc., Santa Cruz, CA, USA). The vehicle was PBS for bradyzide, HOE-140 and WIN 64338, and PBS/0.05% DMSO for ELN-441958. The injection volume was approximately 0.1 ml.

### Mouse shipping and housing

All animal experiments were approved by the National Animal Experiment Board in Finland (ELLA; license numbers ESAVI/2766/04.10.07/2014 and ESAVI/3119/04.10.07/2017) and the Regional State Administrative Agency for Southern Finland (ESAVI) and conducted in accordance with the European Union Directive. We ordered 5-8-week-old DBA/2NCrl (D2) and C57BL/6NCrl (B6) male mice from Charles River Laboratories (Charles River Laboratories, Sulzfeld, Germany). The mice were acclimatised in a temperature (22 ± 2 °C) and humidity (50 ± 15%) controlled facility on a 12-h light/dark cycle (lights on 0600–1800) and single housed at least for 1 week prior to experiments to avoid possible effects of social hierarchy on behaviour and molecular readouts, acknowledging that single housing may influence anxiety-like behaviour of mice.

Behavioural experiments were done in the Mouse Behavioural Phenotyping Facility of Laboratory Animal Centre and in the Rodent phenotyping and *in vivo* experimentation unit of the University of Helsinki, Finland. Prior to any behavioural procedures, mice were transported from the mouse colony room to the experimental room and allowed to habituate for 30 min. If not specified, food and water were available *ad libitum*.

### Restraint stress

Restraint stress was induced by placing the mouse in a 50 ml ventilated Falcon tube for 30–120 min, depending on the experiment, which after tissue samples were collected. In some experiments the mice were allowed to recover for 1–5 h before tissue collection. When restraint stress was applied before behavioural experiments, mice were placed in round (inner diameter 32 mm) acrylic animal holders (Med Associates, Inc., Fairfax, VT, USA) for 30 min which after the mice were allowed to recover for 1 h before behavioural analysis. Vehicle or drug solutions were i.p. injected immediately before restraint stress. The open field test was done after a 1 h recovery period.

### Open field test

Anxiety-related behaviour in the open field test was measured with the use of Activity Monitor system (MedAssociates). For data presented in Fig. 4, the open field arena consisted of a 30 × 30 × 20 cm box with transparent Plexiglas walls and white floor. It was equipped with two rows of infrared light sensors for detection of vertical and horizontal activity. For the data analyses, a 13 × 13 cm centre zone was defined virtually. For data presented in Fig. 5, the open field arena consisted of a 43 × 43 × 30 cm box with transparent Plexiglas walls and white floor. It was equipped with two rows of infrared light sensors for detection of vertical and horizontal activity. Animals were adapted to the experimental room condition with the lighting ~ 150 lx for at least 30 min before the test. The mouse was released in the corner of the open field facing to the wall and allowed to explore the arena for 5 min. The total distance traveled and percent of distance travelled in the centre zone, time spend in the centre zone, the number of entries to the centre and latency to the first centre entry were calculated.

### Forced swim test

The forced swim test was done as previously described^61^. Briefly, after 30 min of adaptation to the experimental room, the mouse was placed in a glass cylinder (diameter = 18 cm, height = 25 cm) filled with 3 litres of water (room temperature) to a height of 15 cm. Time immobile (passive floating) was detected with EthoVision XT10 system (Noldus, The Netherlands) for 6 min with 2 min time bins. Data from the last 4 min was used.

### Elevated plus maze test

The experimental arena consisted of a plus-shaped maze with a central zone (5 × 5 cm), two open (30 × 5 cm) and two closed arms of the same size but with 15 cm high transparent walls. The maze was raised to 38.5 cm above the floor and illuminated with 15–20 lx. The mouse was placed in the centre of the maze facing one of the closed arms and observed for 5 min with the video-tracking system (EthoVision XT10). The total travelled distance and percent of distance travelled in the open arms, time spend in the zones, and the number of entries to the zones were calculated.

### Novelty-suppressed feeding test

Latency to start feeding in a novel environment was measured by EthoVision XT10 software (Noldus) on hungry animals. Animals were deprived of food overnight before the experiment with water available *ad libitum*. For the measurement we put a small plastic cup containing chopped food pellets in the centre of the floor in the white acrylic box (30 × 30 cm, illumination ~150 lx). The mouse was placed in the corner of the box and allowed to explore the arena freely for 5 min or until it started eating the food. We then removed the mouse to its home cage and allowed it to eat the chopped food for 5 min.

### Stress-induced hyperthermia

SIH was done with single-housed mice in the animal room to avoid any transportation stress.

The body temperature was measured at three time points by restraining the mouse in hand by the scruff of the neck and inserting a 1.5 cm long rectal probe for 20 sec. To control for the drug effect on the basal body temperature, the first measurement was taken immediately before the drug injection (T_0_). Animals then received an intraperitoneal injection of WIN 64338 (5 mg/kg) or PBS, followed by the SIH test 60 min later. The SIH test consisted of two measurements of core body temperature with 10 min interval (T_1_ and T_2_). Stress caused by the first measurement results in hyperthermia that was assessed as the difference between the second and the first measurement (T_2_-T_1_).

### Chronic social defeat stress (CSDS)

5-week-old B6 and D2 male mice were subjected to 10-day CSDS as described before^25^. 13–26-week-old male Clr-CD1 mice were used as aggressors. Twenty-four hours after the last day of CSDS, mice were exposed to the social interaction test, and based on their social interaction behaviour they were divided into stress-susceptible (i.e. showing avoidance behaviour) and stress-resilient (i.e. resembling controls) groups as previously described^52^.

### Cold exposure and brown adipose tissue (BAT) dissection

Mice were placed in +4 °C for 4 h. After a 30–40-minute recovery period at room temperature, interscapular BAT was dissected, snap frozen and stored at −80 °C.

### Tissue collection

For mRNA and protein analyses, mouse hippocampus, cortex, hypothalamus and interscapular brown adipose were collected. Tissue samples were snap frozen in liquid nitrogen and stored at −80 °C.

### q-RT-PCR

Total RNA was isolated with TRI Reagent (Molecular Research Center, Cincinnati, OH, USA) and reverse transcribed with iScript Select cDNA Synthesis Kit (Bio-Rad, Hercules, CA, USA). We used the following primer sequences: *Bdkrb1* CCGGTATTGCCCAGTAGGAAAG and GATAGCAGAGACGGTTGGTCAAG; cyclophilin A (*Ppia*): GGAGATGGCACAGGAGGAAA and CCCGTAGTGCTTCAGCTTGAA; *Fos:* GGAATGGTGAAGACCGTGTCA and TCAGGAGATAGCTGCTCTACTTTGC; *Kng1* variants: TGACTGAAATGGCAAGAAGGC and CTGGCAATGTAGGGTGGACTT (variant 1), CTCCTTTCCGGAGTGTCACAG and CCTTTGAGAGTCTGCCCTTGT (variant 2), and AGAAGGCCTCCAGGTTTTTCTC and ATCATGGGCCCCAGTGTCATA (variant 3); *Kng2* variants: GGCTTTTCTCCTTTCCGGTCA and TCTCTGACAATGTAGGGTGGACT (variant 1), AGGCTTTTCTCCTTTCCGGTC and CGCGCGTAGGAGCCTAGT (variant 2), and GGCTTTTCTCCTTTCCGGTCA and GGACATTTCTGGGAAGTTGCTTAG (variant 3); Adrb3^62^: CAACCCGGTCATCTACTG and ACCGTAGCTACACAGAAG; *Glut4*^63^: TTGGCTCCCTTCAGTTTGG and CTACCCAGCCACGTTGCAT; *Pgc1a*^63^: AGACAAATGTGCTTCCAAAAAGAA and GAAGAGATAAAGTTGTTGGTTTGGC; *Ucp1*^62^: CCTGGCAGATATCATCAC and TCACCTTGGATCTGAAGG. 250 ng of DNase I (Thermo Scientific)-treated total RNA was converted to cDNA with iScript select cDNA synthesis kit (Bio-Rad Laboratories). 2–4 µl of 1:10 dilution of the cDNA was amplified with 250 nM primers in CFX384 Real-Time PCR cycler using IQ SYBR Green supermix (Bio-Rad Laboratories) in a total volume of 10 µl. Each reaction was run in triplicate and relative expression level was calculated using a standard curve (10.0, 5.0, 2.0, 1.0, 0.5, and 0.25 ng of cDNA) present on each assay plate with CFX Manager (Bio-Rad Laboratories). The expression levels of genes of interest were normalized to the cyclophilin A housekeeping gene expression levels.

Hippocampus *Bdkrb1*, *Bdkrb2* and cyclophilin A were analysed with PrimePCR Assay (Bio-Rad).

### Western blot

Protein was isolated from frozen homogenized tissue with hot reducing Laemmli sample buffer (Bio-Rad) and analysed by Western blot. The primary antibodies used were anti-BDKRB1 rabbit-IgG (Enzo Life Sciences Inc., NY, USA), anti-BDKRB2 AA 119–131 rabbit IgG (Bioss Antibodies Inc., Woburn, MA, USA), anti-CRYAB clone 731502 (R & D Systems Europe, Abingdon, UK), anti-cFOS SC-52 (Santa Cruz Biotechnology Inc.), anti-HMGB1 IgY^64^ and anti-β-actin clone AC-15 (Sigma-Aldrich). Primary antibodies were detected with peroxidase conjugated secondary antibodies and the bands were visualized with enhanced chemiluminescence (Thermo Fisher Scientific, Waltham, MA, USA). Digital pictures of blots were taken and analysed with ImageJ software^65^. The mean expression level of control samples was defined as 1 and the expression levels of stress samples were normalized to the mean control expression values in each blot. The mean and SEM values of separate Western blots are shown in Fig. 2.

### RNA-sequencing

We dissected the medial prefrontal cortex (mPFC) and ventral hippocampus (vHPC) 6–8 days after the last CSDS, and tissue collection and RNA-sequencing were carried out as described^25^. These data has previously been deposited to Gene Expression Omnibus with accession number GSE109315.

### Statistical analyses

Statistical analysis was carried out with GraphPad Prism v8 (GraphPad software, San Diego, CA, USA) or SPSS (IBM, Armonk, NY, USA). A significance level of P < 0.05 was used to evaluate the null hypothesis. The data were examined using the Student’s *t*-test (unpaired, two-tailed), ANCOVA (covariate analysis) or two-way ANOVA (Bonferroni post-hoc test, Fig. 6b). Differential expression analysis of the RNA-seq data (Fig. 3) is described in^25^ and P-values were corrected for multiple testing using the Benjamini-Hochberg method^66^.

## Supplementary information

Supplemental information

## Data Availability

The RNA-sequencing data analysed during the current study are available through Gene Expression Omnibus with accession number GSE109315. All other data are not publicly available because of the lack of public databases for these data formats but are available from the corresponding author on reasonable request.
