# Supplementary material for: The bradykinin system in stress and anxiety in humans and mice
Source: Sci Rep. 2019 Dec 19;9:19437. doi: 10.1038/s41598-019-55947-5 (PMC6923437; doi:10.1038/s41598-019-55947-5)
Supplement: Supplementary file 1 — Supplemental information [file 41598_2019_55947_MOESM1_ESM.pdf]

The bradykinin system in stress and anxiety in humans and mice

Ari Rouhiainen[1,*], Natalia Kulesskaya[1,*], Marie Mennesson[1, 2, 3, 4], Zuzanna Misiewicz[1, 2], Tessa Sipilä[1], Ewa Sokolowska[1], Kalevi Trontti[1, 2, 3, 4], Lea Urpa[1], William McEntegart[1], Suvi Saarnio[1], Petri Hyytiä[5], Iiris Hovatta[1, 2, 3, 4]

[1] Molecular and Integrative Biosciences Research Program, University of Helsinki, Helsinki, Finland

[2] Department of Psychology and Logopedics, Medicum, University of Helsinki, Helsinki, Finland

[3] SleepWell Research Program, Faculty of Medicine, University of Helsinki, Finland

[4] Neuroscience Center, Helsinki Institute of Life Science HiLIFE, University of Helsinki, Finland

[5] Department of Pharmacology, Medicum, University of Helsinki, Helsinki, Finland

Supplemental information:

Supplementary Table 1

Supplementary Figure 1

Supplemental Table 1. Genotyped SNPs. The SNP was not analyzed for association if it failed quality control.

Gene	SNP	Reason for failing quality control
BDKRB1	rs4905475	
	rs10134118	Not in Hardy-Weinberg equilibrium
	rs11622768	
	rs10147171	
	rs10137303	
	rs11628515	
	rs10143977	Genotyping failed
	rs2081084	Genotyping failed
	rs10142830	
	rs885845	
	rs8010789	
	rs945036	
	rs4349080	
	rs4905476	
	rs11160324	Genotyping failed
	rs7147284	
	rs11621078	
	rs1959049	
	rs945034	
	rs2369523	
	rs2069613	
BDKRB2	rs945032	
	rs8016905	
	rs1046248	
	rs4900312	
	rs4900313	
	rs945039	
	rs4905467	
	rs11847625	Genotyping failed
	rs8013400	
	rs8012552	
	rs4905470	
	rs7149163	
	rs2069575	
	rs2069578	
	rs5225	
	rs2069590	Genotyping failed
	rs2069596	
	rs2069599	Genotyping failed
	rs4900311	
	rs11160319	
	rs11625405	
	rs1799722	
	rs1959053	
	rs12433275	Genotyping failed
KNG1	rs7624355	Genotyping failed
	rs13315296	
	rs11918289	
	rs11924390	
	rs1624230	
	rs1621816	
	rs1469859	
	rs1656922	
	rs2304456	
	rs5030028	
	rs5030102	
	rs266723	
	rs5030039	
	rs1624569	
	rs5030060	
	rs710446	
	rs5030091	
	rs266760	
	rs266763	
	rs1972703	
	rs266764	
	rs6796803	

Supplemental Figure 1: Uncropped Western blots of Figure 2.

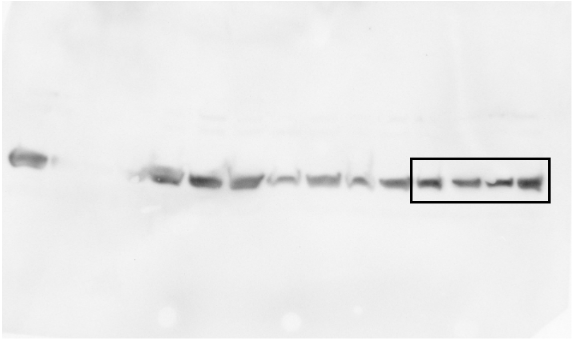

Fig. 2a BDKRB2

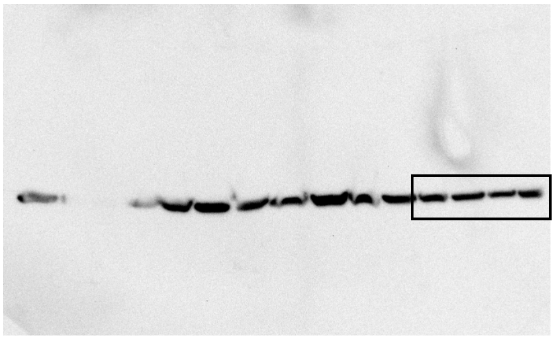

Fig. 2a Actin

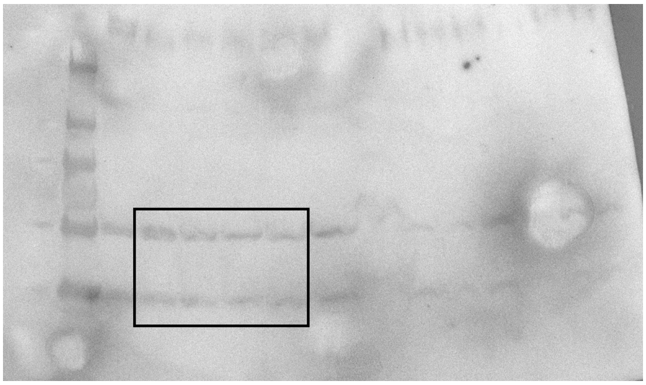

Fig. 2b BDKRB1

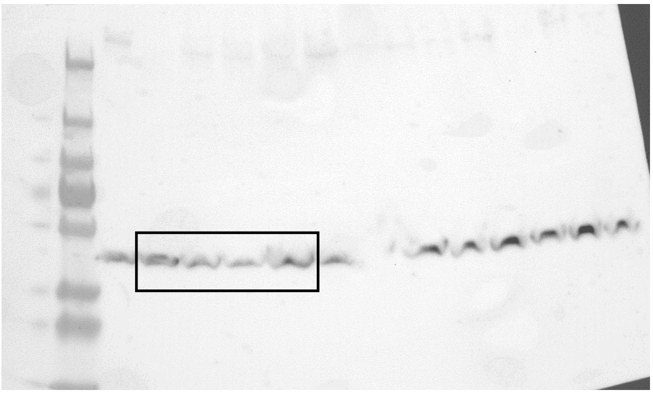

Fig. 2b Actin

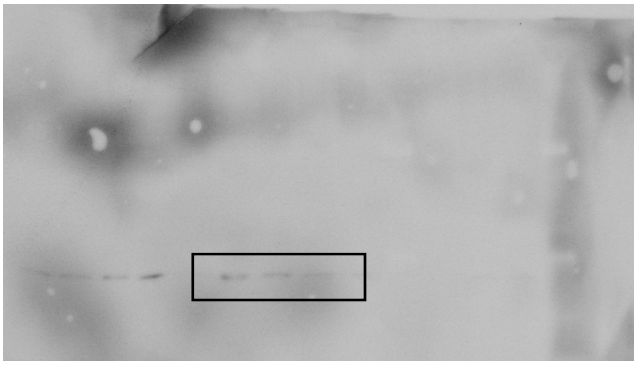

Fig. 2c CRYAB

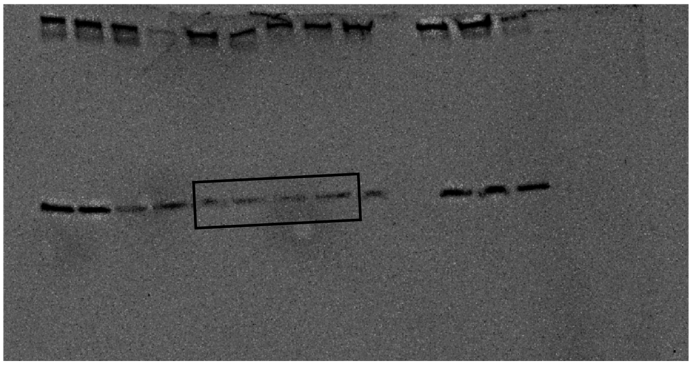

Fig. 2c Actin

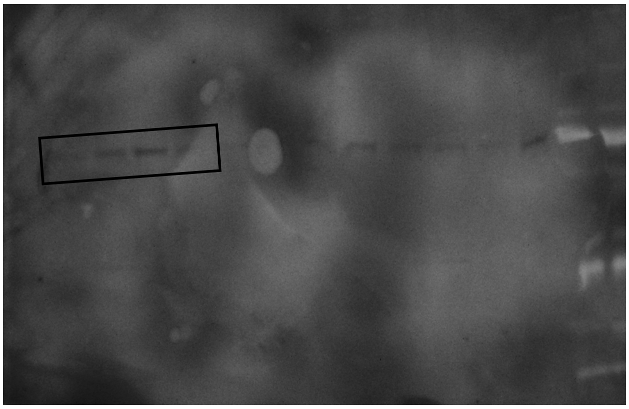

Fig. 2d cFOS

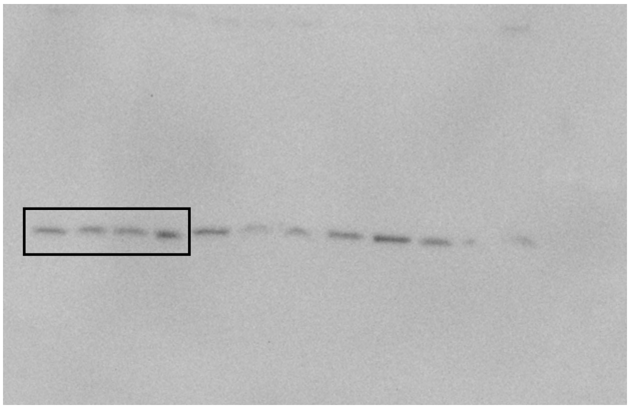

Fig. 2d Actin

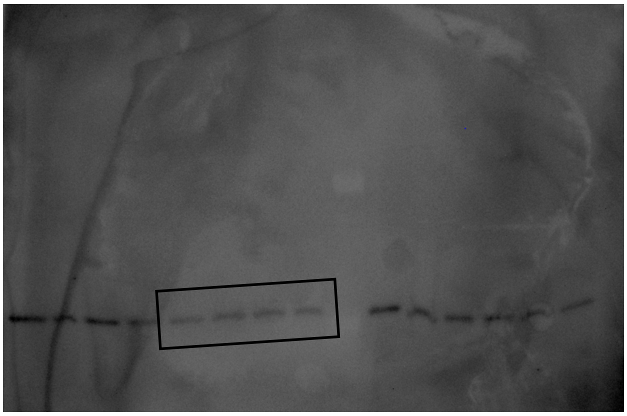

Fig. 2e HMGB1

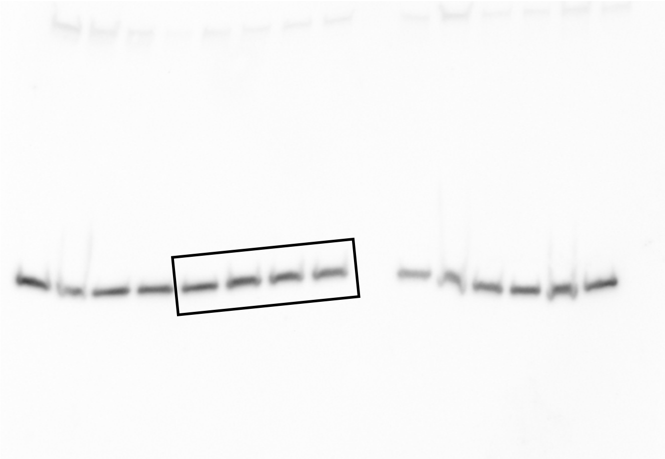

Fig. 2e Actin
